# Congenital ureterovaginal fistula: a rare case of single-system ectopic ureter with ipsilateral ectopic kidney managed by vaginal approach: a case report

**DOI:** 10.1186/s13256-021-03157-x

**Published:** 2021-12-15

**Authors:** Demisew Amenu, Andebet Asmare, Ahmed Siraj

**Affiliations:** Department of Obstetrics and Gynecology, Jimma University, Jimma, Ethiopia

**Keywords:** Ectopic ureter, Ureterovaginal fistula, Single system, Congenital

## Abstract

**Background:**

Diagnosing urinary incontinence from organic causes such as ectopic ureter is particularly important because of the potential for cure by surgical correction. The prevalence of ectopic ureter is uncertain because many are asymptomatic and the diagnosis is usually overlooked. Eighty percent of ectopic ureters in females are often associated with duplex kidney. However, an ectopic ureter draining a single-system ectopic dysplastic/atrophic but functioning kidney is rare, especially in females. The overall long-term continence rate after successful correction of ectopic ureter is satisfactory.

**Case presentation:**

This case is reported to highlight a rare situation, where a 22-year-old nulligravid Ethiopian women presented with a complaint of continuous wetting of her underwear since childhood, but she had normal voiding pattern. Localized right pelvic kidney ultrasound and computed tomography scan with contrast revealed right ectopic ureter and atrophied ipsilateral pelvic kidney with good function. Surgical reimplantation through vaginal approach was performed, and the outcome was good. The patient’s subsequent follow-ups were uneventful.

**Conclusion:**

An extramural vaginal ectopic ureter is better accessed through transvaginal approach than abdominal, especially when it is associated with pelvic ectopic kidney. This modified approach is less invasive and has lower morbidity and better success rate than a transabdominal approach.

## Introduction

Urinary incontinence caused by functional disturbances is a common problem during childhood, which makes the treatment outcome unsatisfactory. However, incontinence from organic causes such as ectopic ureter (EU) is particularly important because of the potential for cure by surgical correction [1]. Ectopic ureter is defined when it opens into a region apart from the usual place on the trigone of the bladder [2]. Embryologically, at approximately the fifth week of fetal life, the ureteric bud arises from the mesonephric (Wolffian) duct. An ectopic ureter forms when the ureteric bud arises abnormally cephalad to the mesonephric duct or when separation between the two is delayed or absent [3]. Renal maldevelopment is commonly associated with ectopic ureter when the ureteric bud has abnormal, associated with metanephric blastema [4]. The prevalence of EU is uncertain because many are asymptomatic and the diagnosis is usually overlooked [2, 5]. Female-to-male ratio is 2–6:1 and in both sexes it may present as a prenatal diagnosis of hydroureteronephrosis [3, 6].

Eighty percent of ectopic ureters in females are often associated with duplex kidney. However, ectopic ureters draining a single-system ectopic ureter with dysplastic kidneys are rare, especially in females [1, 5, 6].

Diagnosis is made by imaging studies that identify the ectopic ureter and associated anomalies. Ultrasound (US) is the first imaging modality that shows a dilated ureter down to its course and hydronephrotic collecting system. However, US may not be useful in single-system ectopia where the kidney may be dysplastic and difficult to visualize. In this case, magnetic resonance imaging (MRI) and computed tomography (CT) give a better delineation of the ectopic kidney and non-dilated upper pole ureter draining ectopically [4, 7]. A renal scan is used to assess renal function prior to surgery.

Gibson incision and ventral midline celiotomy with ventral longitudinal cystectomy and proximal ureterectomy are commonly used approaches employed during surgery. The choice of surgical techniques for correction of a single-system EU depends on the degree of renal function, associated anomalies, and the location of the EU (whether it is intramural or extramural). Nephroureterectomy is indicated when the kidney is dysplastic and its function is compromised (function of less than 10%) with normal contralateral kidney. When the kidney function is preserved, ureteric reimplantation is the surgery of choice not only to restore the continence, but also to spare the function of the remaining nephrons. Neoureterostomy and urethral-trigonal reconstruction are indicated to repair intramural ectopic ureters, whereas neoureterocystostomy is performed to repair an extramural ectopic ureter [8–10]. The overall long-term continence rate after successful correction of EU reaches up to 81%. This continence rate can be improved by adjunct medical treatment, consisting mainly of α-adrenergic agents and application of colposuspension to treat stress incontinence due to urethral sphincter abnormalities, which are one of the major causes of recurrence of urinary incontinence (UI) after surgery [11, 12].

## Case presentation

**Fig. 1:**
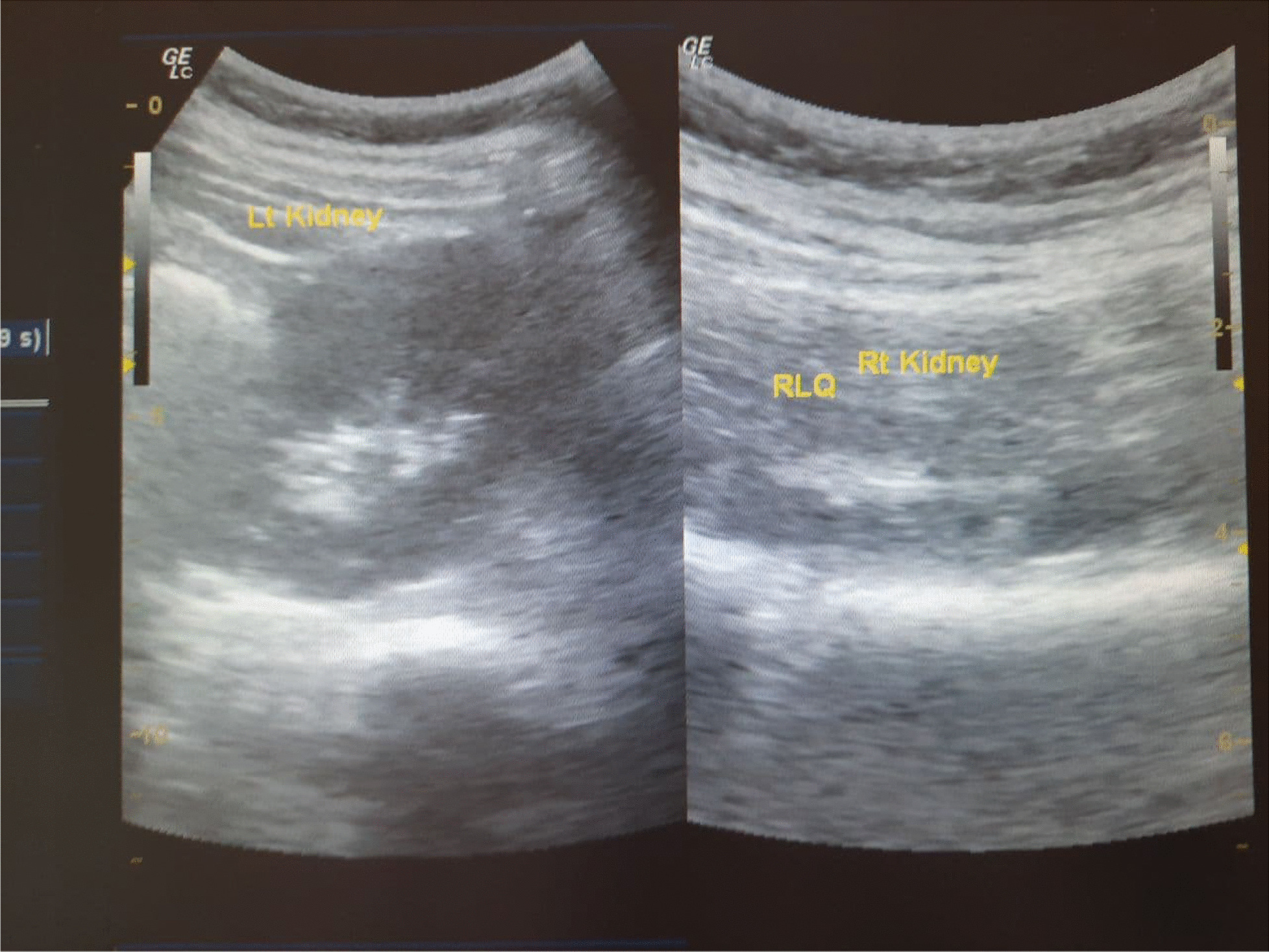
Ultrasound of the right pelvic atrophic kidney and left normal kidney before surgery

**Fig. 2:**
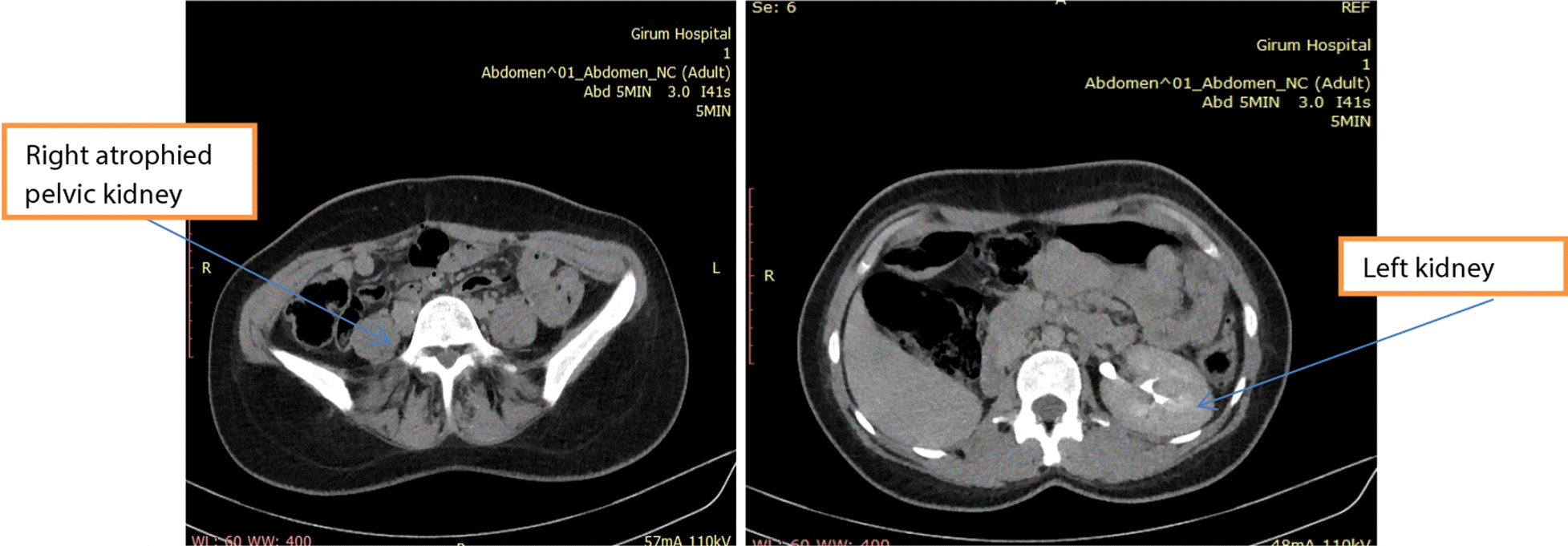
CT scan with contrast shows the right atrophied pelvic kidney and left normal kidney before surgery

**Fig. 3:**
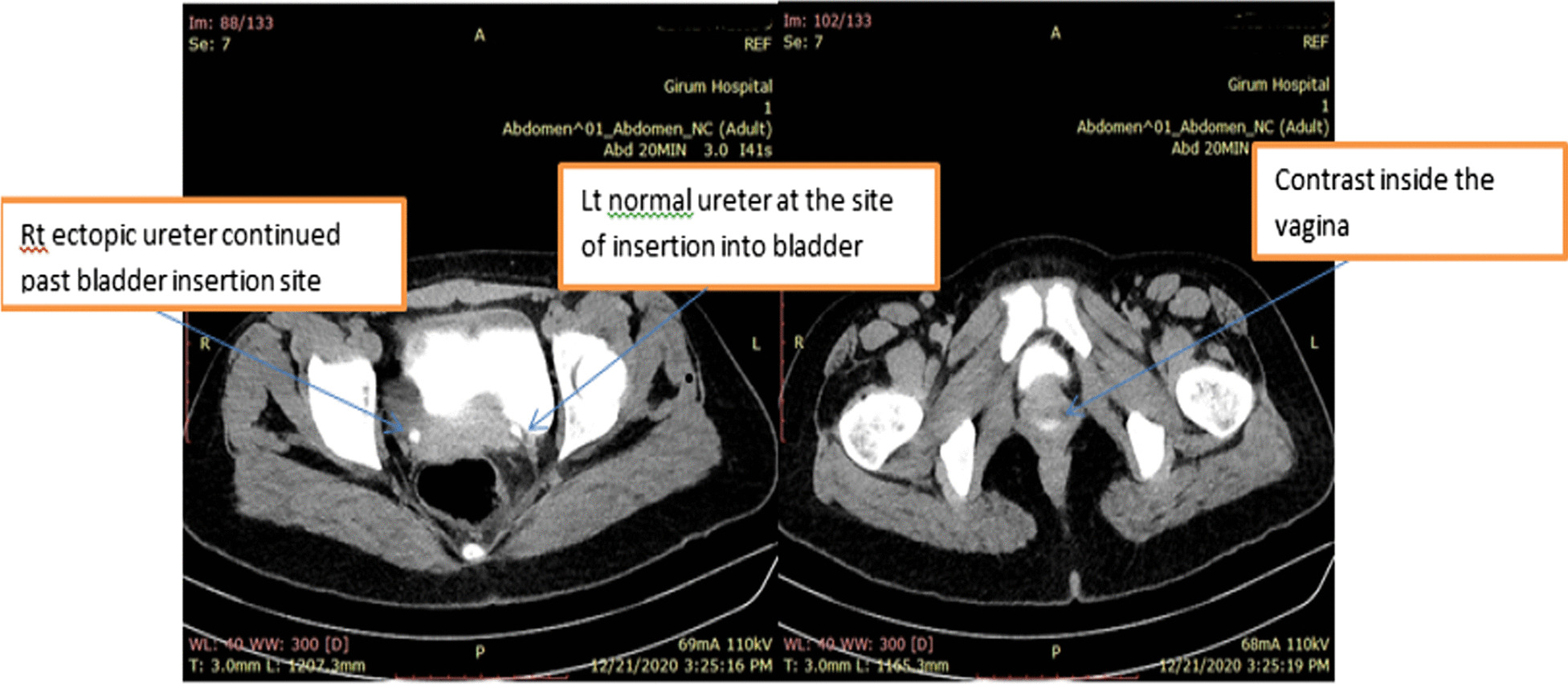
CT scan with contrast image shows right ectopic ureter and left normal ureter at site of bladder insertion (left side) and contrast tracking into the vagina (right side)

**Fig. 4:**
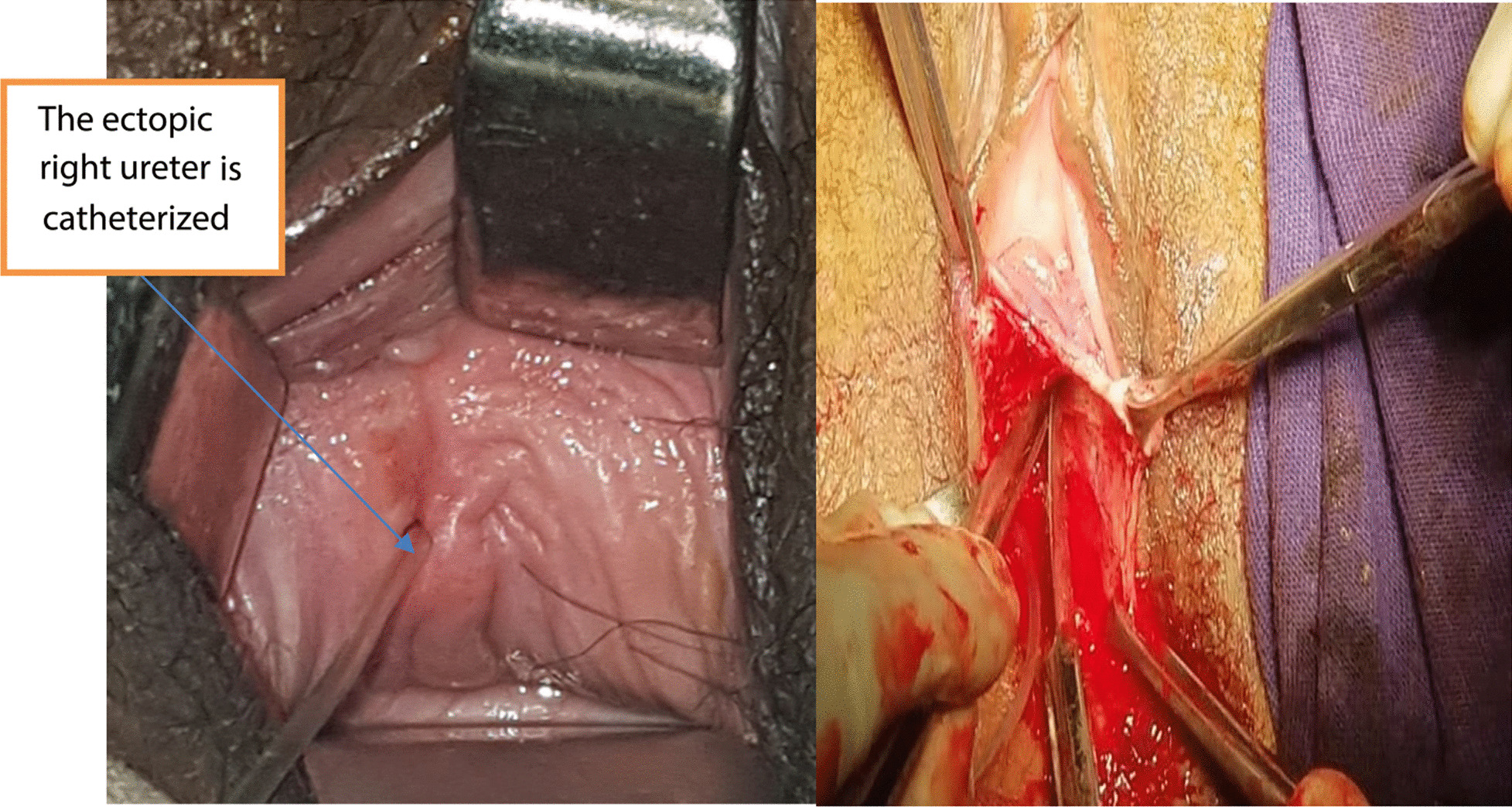
The right ectopic ureter is catheterized (Left) and the vaginal mucosa dissected of the ectopic ureter (right)

A 22-year-old nulligravid Ethiopian woman came to the Jimma University Medical Center (JUMC) outpatient department with a complaint of continuous wetting of her underwear since childhood, but she had normal voiding pattern. She visited our hospital twice, 6 years ago for the same complaint, but she was sent home with reassurance and advice to do Kegel exercises. She had no any previous history of pelvic surgery, trauma, lower urinary tract symptoms, or treatment for urinary tract infections. She had no history of previous known chronic medical illness. On physical examination of the genitourinary system, she had no costovertebral angle tenderness, her perineum was wet, and there was pooling of urine in her vagina. A small pinpointed hole was seen on the right anterolateral aspect of vaginal wall, with spurting of urine upon catheterization with a 6 Fr pediatric nasogastric tube NGT) (Fig. 4). We inserted a foley catheter into the bladder and instilled with 60 cc methylene blue dye to rule out congenital vesicovaginal fistula, and the dye test was negative. Pelvic ultrasound localized the left normal kidney in place and the right atrophic kidney in the pelvis (Fig. 1). The bladder had normal capacity. Uterus and ovaries appear normal. With a suspicion of ectopic ureter, a CT scan with contrast was done and revealed right ectopic ureter and atrophied right pelvic kidney with intermittent ureteral stricture, whereas the contralateral kidney and ureter appear normal (Figs 2 and 3). MRI and renal scintigraphy are not available in our institution. Complete blood count, renal function test, and urinalysis were all normal.

**Fig. 5:**
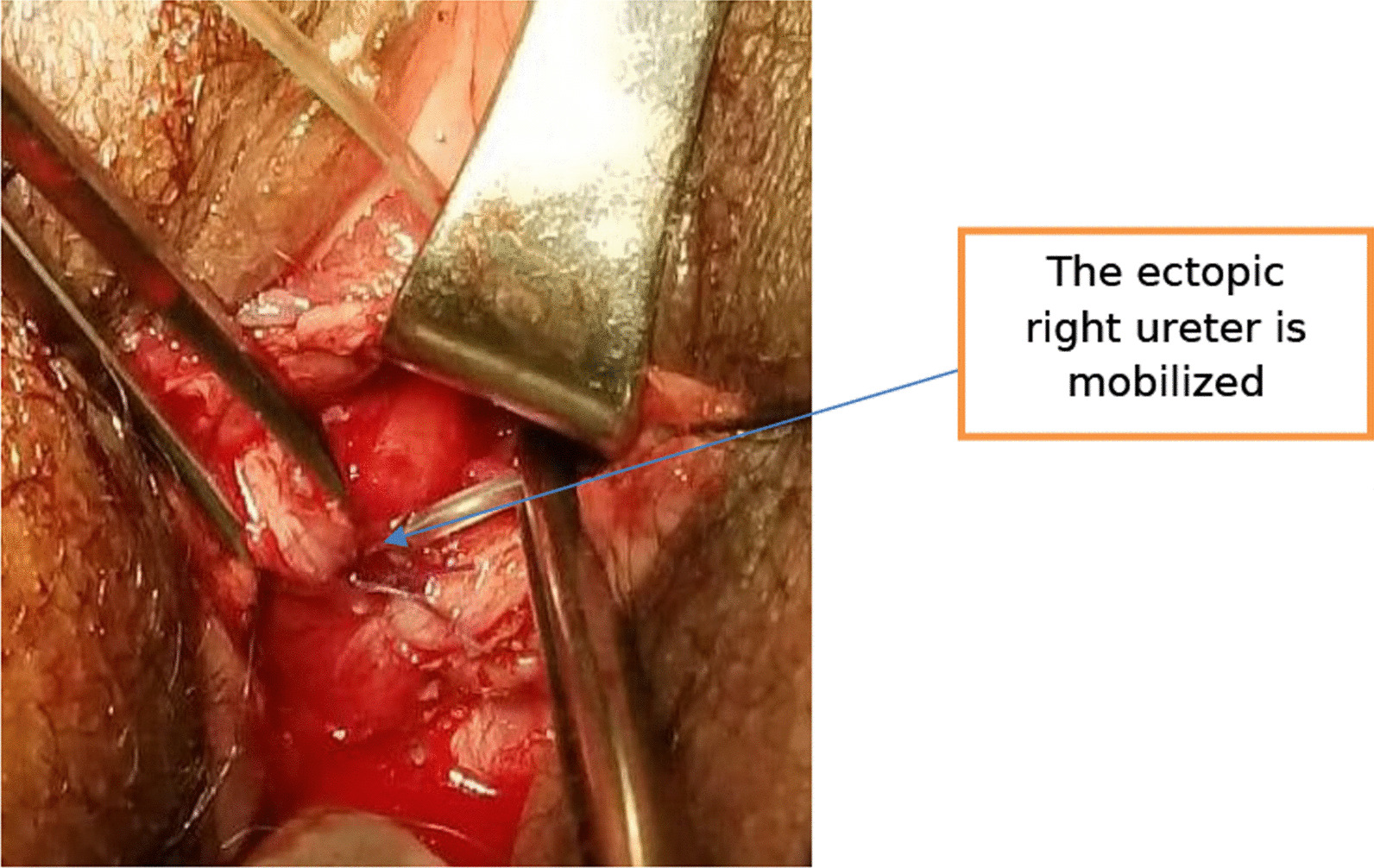
The ectopic ureter is mobilized from the right side of vagina, cystotomy was made at mid-vagina and the Fr 6 pediatrics NGT exteriorized through the urethra

**Fig. 6:**
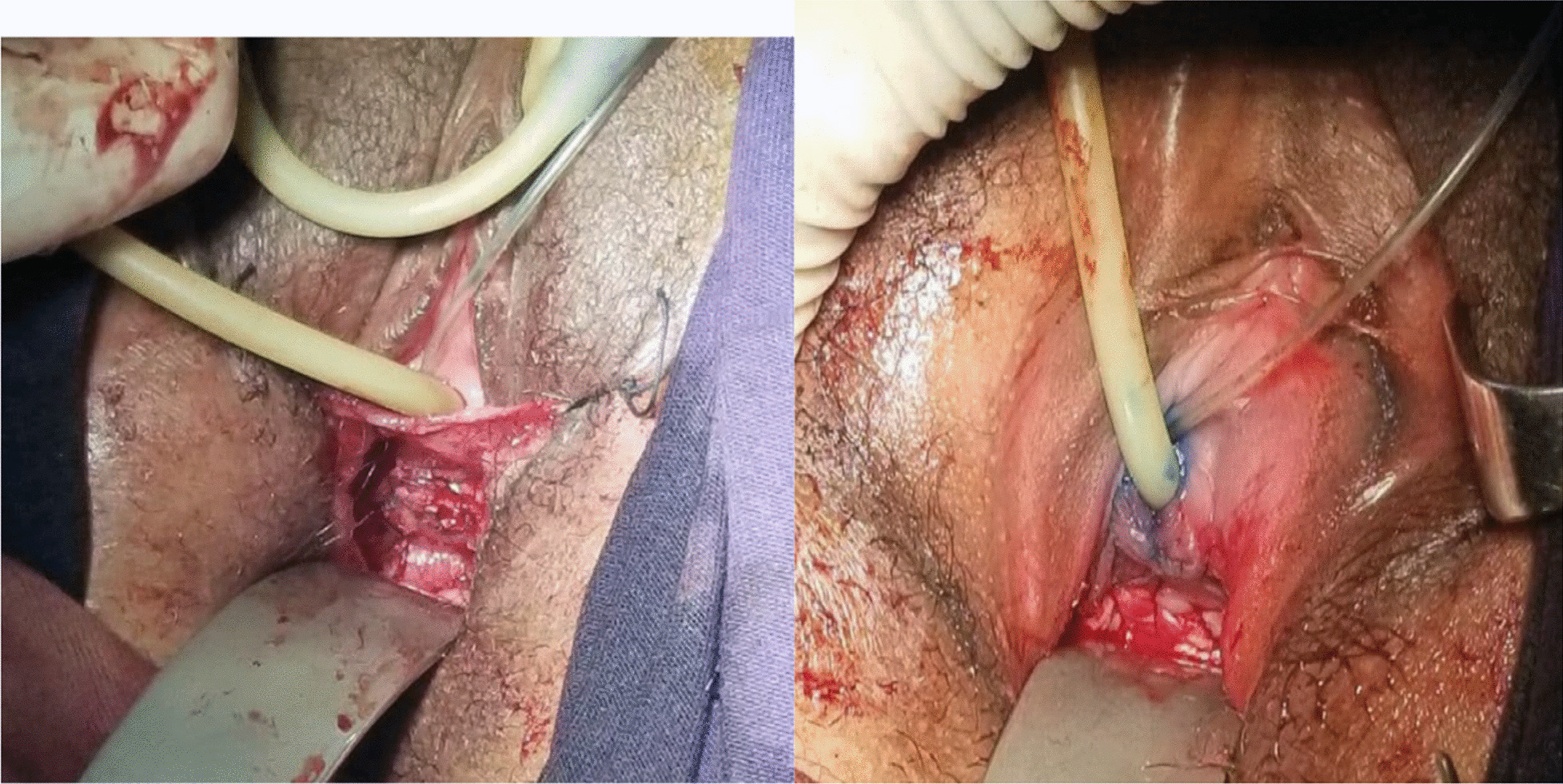
The ectopic ureter is re-implanted, the bladder closed in layers, and dye test was performed (left), The vaginal mucosa was closed with Vicryl no. 2-0 and both ureteral and foley catheters put in place (right)

Because the right kidney is pelvic with a good function, we decided to approach the surgery through the vagina. The patient was given spinal anesthesia and put in a dorsal lithotomy position. After making a circumscribing incision around the ectopic ureter, the ureter was mobilized from the bladder and vagina, and catheterized with Fr 6 pediatric NGT. Cystotomy was done at mid-vagina close to the ectopic ureter, and the ureter was re-implanted to the bladder at the trigone without tension using Vicryl # 3-0. After the ureteric catheter was exteriorized through the urethra, the bladder was closed in two layers using Vicryl # 3-0. Finally, the dye (methylene blue) test was repeated intraoperatively to check for water tightness and when it became negative the vaginal mucosa was closed (Figs 4, 5, 6). The patient was transferred to the gynecology ward with the plan of keeping the ureteric catheter for 2 weeks and the bladder catheter for 7 days.

**Fig. 7:**
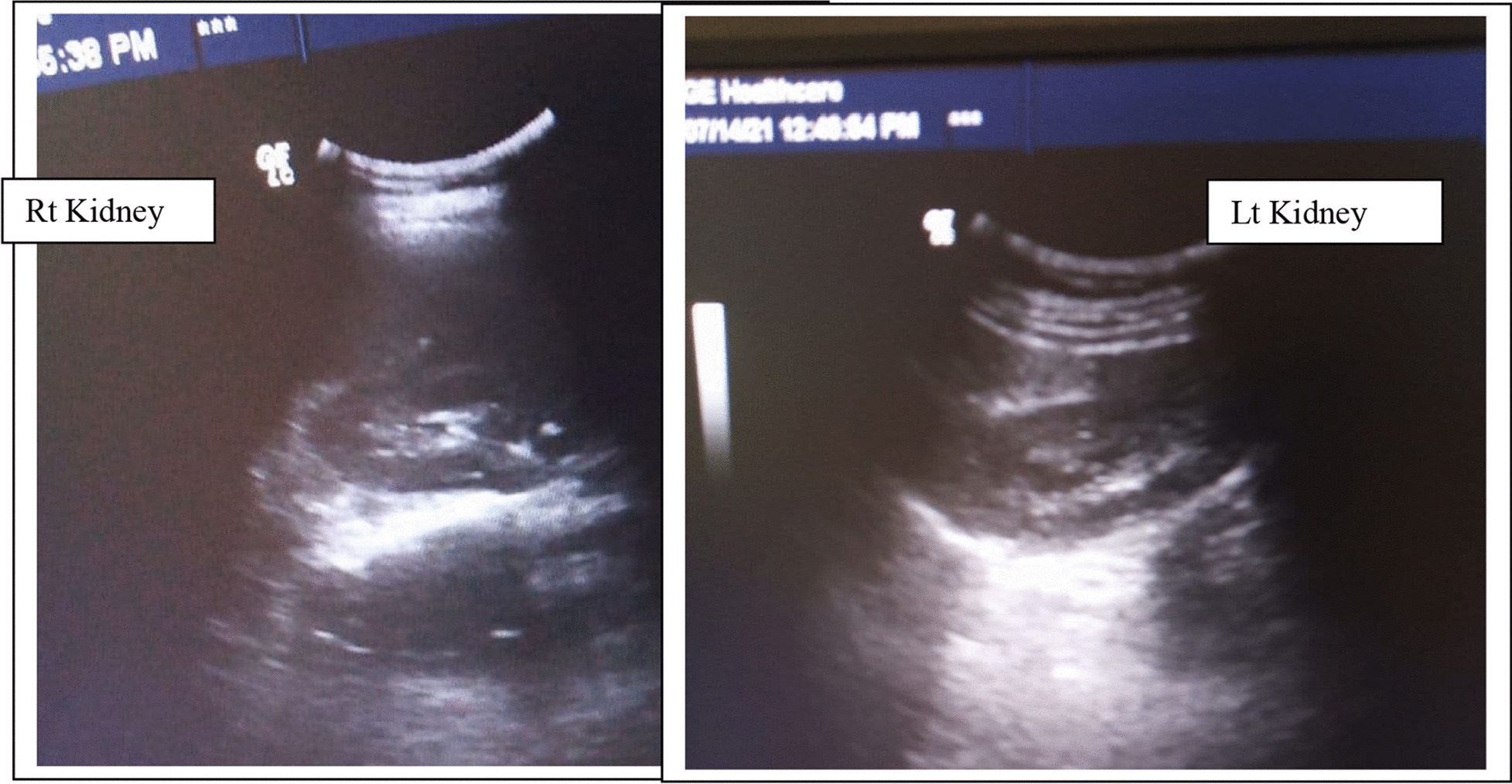
US of the right and left kidneys 1 month after surgery

After 7 days of surgery, the bladder catheter was removed and the patient was continent. Then, the ureteric catheter was also removed after 2 weeks. The ultrasound of the right kidney was normal and the patient was discharged with an appointment. She had two follow-ups at 1 and 3 months after surgery. All follow-up imaging and renal function tests showed normal findings. Figure 7 shows the ultrasound image of the kidneys 1 month after surgery (Fig. 7). She reported no difficulty in voiding, or pain or recurrent urinary tract infections until her last visit.

## Discussion

Commonly, the diagnosis of EU is made during childhood owing to recurrent urinary tract infections or urinary incontinence [3, 6]. However, a single-system ectopic ureter is a diagnostic challenge and is often delayed, especially when it is associated with a small dysplastic, poorly functioning, non-visualized kidney, which may be difficult to see even with conventional imaging studies and having a contralateral normal solitary kidney [1, 13]. Our patient came to visit the health facility during adulthood and the diagnosis was delayed because of improper evaluation. In males, an ectopic ureter is always inserted proximal to external urethral sphincter, and they are usually continent. In females, the ureteral opening may be located in the urethra, in the vagina, in the uterus, or in the fallopian tubes along the mullerian duct structures that bypass the urethral external sphincter [3]. Twenty-five percent of ectopic ureter open into the vagina. So girls present with small volume of leakage or spotting but they have normal voiding patterns. Our patient presented with the same complaint since childhood. The diagnosis of EU in our patient was made clinically by a negative dye test and inspection of a small unilateral hole with spurting of urine in the right lateral vaginal wall, through which advancing an infant feeding tube is possible. It was confirmed by CT urography, which also showed atrophic ipsilateral pelvic kidney in addition to the EU. A single-system ectopic ureter (SSEU) is rare in females and commonly associated with other anomalies such as in this patient where it was associated with ipsilateral ectopic kidney [7]. Surgery of an SSEU depends on the degree of renal function, associated anomalies, and the location of the EU [5]. In single-system ectopic ureter, the affected kidney is usually small and poorly functional and the management is nephroureterectomy (removal of the kidney and ureter) [5]. Preservation of the renal function in the affected kidney makes our case unique and increases the difficulty in decision-making of the management. If the renal function is good and the kidney is not dysplastic, the most important factor that affects the surgery is the location of the EU. An intramural ectopic ureter is found attached to the serosal surface of the bladder but fails to terminate and open into the bladder lumen at the trigone. Instead, it continues to tunnel through the trigone in the submucosa to open at a site distal to the bladder neck. In this case, UI may result not only from the ectopic position, but also from disruption of the smooth muscle of urethral sphincter mechanism by the submucosal ureteral tunnel. This is managed surgically by neoureterostomy and urethral-trigonal reconstruction. Whereas, repair of an extramural ectopic ureter is done by ureteral reimplantation (neoureterocystostomy). The goal of surgery is to restore the ureteral orifice to a position proximal to the urethral sphincter, thereby restoring urinary outflow control [8]. In our case, the kidney was small, ectopic, and had good function. So, we preserved the kidney and re-implanted the ectopic ureter to the bladder wall at the bladder trigone. Grover *et al.* reported a case of right EU terminating into the right lateral wall of the vagina, with good function of the right kidney managed by right ureteric re-implantation (extravesical ureteroneocystostomy). In this case, follow-up of the patient after 3 months was uneventful [9]. The management of this case is similar to our case. But, in our case, the ectopic pelvic kidney opening into the lateral vagina made a transvaginal surgical approach easier.

This surgery decreases the risk of recanalization of the distal segment because we mobilized the ureter at its most distal segment. This approach is also important in patients having ipsilateral ectopic kidney with good function because it decreases the postoperative morbidity and invasive abdominal or inguinal surgery.

## Conclusion

Although it is common to find studies on female girls suffering from congenital urinary incontinence due to ectopic ureter, it is very rare to find SSEU having a functioning ectopic ipsilateral kidney. An extramural vaginal ectopic ureter is better accessed through a transvaginal approach than abdominally, especially when it is associated with pelvic ectopic kidney. In conclusion, for girls with a compliant of any degree of urinary incontinence or wetting, a thorough evaluation to rule out congenital vesical fistula and EU has to be the first step.

This study suggests that this modified surgery has advantages of easy access, decreased morbidity, and successful surgical outcome compared with routine abdominal surgeries if the fistula opens into the vagina.

## Limitation of the study

There are no studies that describe about long-term outcome of this surgery. The function of the involved kidney is not assessed with renal scintigraphy because of the limitation of resources in our institution.

## Data Availability

Data sharing is not applicable to this article as no datasets were generated or analysed during the current study.

## Abbreviations

EU: Ectopic ureter
US: Ultrasound
MRI: Magnetic resonance imaging
CT: Computed tomography
UI: Urinary incontinence
JUMC: Jimma University Medical Center
NGT: Nasogastric tube
SSEU: Single-system ectopic ureter

## References

[1] Gangopadhyaya AN, Upadhyaya VD, Pandey A, Gupta DK, Gopal SC, Sharma SP, et al. Single system ectopic ureter in females: a single center study. J Indian Assoc Pediatric Surg. 2005;12:202–5.

[2] Demir M, Çiftçi H, Kılıçarslan N, Gümüş K, Oğur M, Gülüm M, et al. A case of an ectopic ureter with vaginal insertion diagnosed in adulthood. Turkish J Urol. 2015;41(1):2014–6. 10.5152/tud.2014.81567 PMC4548654 26328201

[3] Baskin LS, Wilcox D, Kim MS. Ectopic ureter. Up to date; 2016. http://www.uptodate.com/contents/ectopic-ureter.

[4] Shortliffe LMD. A case of ectopic dysplastic kidney and ectopic ureter diagnosed by MRI; 2014. 10.1038/ncpuro1220 18839014

[5] Basavaraju M, Zachariah N. Solitary ureteric ectopia with incontinence: a case report and review of literature. J Curr Res Sci Med. 2016;2:39–41.

[6] Duicu C, Kiss E, Simu I, Aldea C, Reed FJ. A rare case of double-system with ectopic ureteral openings into vagina. Front Pediatrics. 2018;6:1–5. 10.3389/fped.2018.00176 PMC6020766 29974045

[7] Dange AS, Sen S, Zachariah N, Chacko J, Mammen KE. Associated malformations and management in children lacking an orthotopic ureter. Pediatr Surg Int. 1994;9:377–80.

[8] Jerram RM, Diplomate ACVS. The piddling puppy—management of ectopic ureter. 2004; 83–5.

[9] Grover JK, Soni DK, Khan S. Successful management of single system ectopic ureter with preserved renal function in a female child: a case report. Int Surg J. 2021;8(3):1033–5.

[10] Demirtas T, Tolga S, Golbasi A, Sonmez G. Urology Case Reports The ectopic ureter opening into the vulva, which is a rare cause of lifelong urinary incontinence: treatment with ureteroureterostomy. Urol Case Rep. 2021;36:101597. 10.1016/j.eucr.2021.101597. PMC7881214 33614412

[11] Noel SM, Claeys S, Hamaide AJ. Surgical management of ectopic ureters in dogs: Clinical outcome and prognostic factors for long-term continence*. Vet Surg. 2017;46:631–41. 10.1111/vsu.12654 28390102

[12] Jeong IS, Rahman M, Kim H, Kim S. Surgical management of extramural ectopic ureter by modified colposuspension following ureteroneocystostomy in a young female Siberian Husky dog. J Adv Vet Animal Res. 2017;4:301.

[13] Chowdhary SK, Lander A, Parashar K, Corkery JJ. Single—system ectopic ureter:a 15-year review. Pediatr Surg Int. 2001;17:638. 10.1007/s003830100011 11727057

